# *Strongyloides stercoralis* in Alcoholic Patients: Implications of Alcohol Intake in the Frequency of Infection and Parasite Load

**DOI:** 10.3390/pathogens9060422

**Published:** 2020-05-28

**Authors:** Joelma N. de Souza, Cíntia de L. Oliveira, Wéslei A. C. Araújo, Alex B. S. Souza, Mônica L. S. Silva, Irlana D. R. da Cruz, Larissa M. Sampaio, Juliane S. B. dos Santos, Márcia C. A. Teixeira, Neci M. Soares

**Affiliations:** Faculdade de Farmácia, Universidade Federal da Bahia, Salvador, Bahia 40170115, Brazil; joelmandesouza@gmail.com (J.N.d.S.); cintia.lima@bol.com.br (C.d.L.O.); wesleicosta11@hotmail.com (W.A.C.A.); brunopharmacia@gmail.com (A.B.S.S.); monica_lopes10@hotmail.com (M.L.S.S.); irlana-dias@hotmail.com (I.D.R.d.C.); sampaiomotalarissa@gmail.com (L.M.S.); julianesilvab15@hotmail.com (J.S.B.d.S.); marciat@ufba.br (M.C.A.T.)

**Keywords:** *Strongyloides stercoralis*, alcoholic individuals, parasite load

## Abstract

*Strongyloides stercoralis* infection in immunocompromised subjects, including chronic alcoholics, can lead to a severe disease. Moreover, its prevalence in alcoholic patients seems to be higher than that in the general population. The aims of this study were to evaluate the frequency of *S. stercoralis* infection in alcoholic patients and to investigate the influence of alcohol intake on the parasite load, as well as to evaluate the sensitivity of three different parasitological methods according to the larval output. Fecal samples of 1290 chronic alcoholic patients were examined by spontaneous sedimentation, Baermann–Moraes, and agar plate culture (APC) methods. *S. stercoralis* was the most frequent parasite found (14.5%; n = 187). Alcoholic individuals infected with *Strongyloides stercoralis* had a higher daily consumption of alcohol than those who were not infected, 528.6 and 403.0 g/day, respectively (p < 0.05). In addition, individuals with higher alcohol intake presented an increase in parasite load. The *S. stercoralis* diagnostic method with the highest sensitivity was APC, 97.9% (183/187). In conclusion, *S. stercoralis* seems to be the most frequent parasite found in alcoholic individuals from endemic areas and alcohol intake is positively associated with *S. stercoralis* larvae output. In addition, this study confirms that APC is the most sensitive parasitological method used for *Strongyloides* diagnosis.

## 1. Introduction

*Strongyloides stercoralis* has a worldwide distribution. Nevertheless, epidemiological information on strongyloidiasis is relatively scarce, and the disease is severely underestimated, mainly due to a limited number of studies and suboptimal diagnostic methods [1,2]. It is estimated that approximately 370 million people are infected worldwide, with the majority residing in tropical and subtropical regions [3]. However, the increasing number of migrant populations from endemic areas—coupled with a larger number of clinical conditions that impair the host immune system—has highlighted *S. stercoralis* infection as one of the most neglected and emerging worldwide infectious diseases [2,4].

Unique among nematodes that infect humans, along with *Capillaria* subspecies, rhabditiform larvae of *S. stercoralis* can transform into invasive filariform larvae while inside the host, before being excreted. This leads to a reinfection by the larvae invasion of the intestinal wall [5] and is called autoinfection. When it occurs in low, well-regulated levels, it leads to chronic infection in immunocompetent individuals [6]. However, in immunocompromised patients, the autoinfection cycle can be intensified, leading to hyperinfection and/or dissemination of strongyloidiasis, which can potentially be life-threatening and has a mortality rate of up to 87% [7,8]. Various drug exposures and clinical conditions that impair the host immune response have been reported to predispose to severe strongyloidiasis, such as corticosteroid use, HTLV-1 coinfection, and alcoholism [5]. 

The prevalence of *S. stercoralis* infection in alcoholic patients is reported to be approximately five times higher than that in the general population [9,10,11]. One possible mechanism includes an alteration in the hypothalamic–pituitary–adrenal (HPA) axis function, raising the levels of endogenous corticosteroids and its metabolites, which resemble an ecdysteroid hormone that regulates the fertility of *S. stercoralis* parthenogenetic females, induces the transformation of rhabditiform to infective filariform larvae, and increases the autoinfection rate [11]. A recent study by our group has demonstrated that increased endogenous cortisol levels is directly related to parasite load [10]. Moreover, chronic alcohol consumption also has a toxic effect on the contractile proteins of the small intestine muscle and on the vagal function, reducing the gastrointestinal transit and favoring the rhabditoid larvae permanence in the host intestinal lumen long enough to undergo ecdysis, which also enhances the autoinfection cycle [12,13]. 

To prevent *S. stercoralis* subclinical infections from developing into severe strongyloidiasis, it is necessary to test high-risk individuals using reliable diagnostic methods. Definitive diagnosis still relies on detection of larvae in the stool; however, this method of detection is challenging since the larvae output in the stool is usually low and intermittent [2]. Parasitological sensitivity can be improved with the analyses of several samples, reaching almost 100% when seven fecal samples are studied [14]. However, it is impractical for patients to return to the laboratory to deliver several stool samples. In this sense, serology offers an alternative to parasitological examination, as it relies on the detection of specific antibodies in sera and does not depend on the intensity of larva excretion in stools [2,5]. Despite their utility, these antibody-based immunoassays have several limitations, which include difficulties in antigen production, cross-reactivity in patients with other helminth infections, lower sensitivity in immunocompromised patients, and an inability to distinguish between current and past infections [5]. Indeed, in a recent study published by our laboratory, it was demonstrated that alcoholic patients presented a lower production of IgG1 and IgE anti-*S. stercoralis* antibodies [15].

The aim of this work was to evaluate *S. stercoralis* frequency in alcoholic patients and to correlate the alcohol intake with both parasite load and sensitivities of three different parasitological methods (spontaneous sedimentation, Baermann–Moraes, and agar plate culture).

## 2. Materials and Methods

### 2.1. Patients

The present study was carried out from September 2012 to December 2018 in alcoholic patients seen at the Alcoholic Care and Treatment Center (Centro de Acolhimento e Tratamento de Alcoolistas—CATA) of the Charitable Works Foundation of Sister Dulce (Obras Sociais Irmã Dulce—OSID), a non-governmental organization in Salvador, Bahia, Brazil, supported by the National Health System. All CATA’s patients were seen by a multidisciplinary team and underwent a clinical, psychiatric, psychological and nutritional assessment. They were diagnosed as alcoholics according to a set of behavioral, cognitive, and physiological phenomena that were developed after repeated use of alcohol. These phenomena are typically associated with the following symptoms: a strong desire to drink (unable to stop drinking after starting), a continued pattern of excessive drinking despite the negative consequences, a need for larger doses of alcohol to achieve the same effect, and sometimes, a state of physical withdrawal (symptoms, such as sweating, tremors, and anxiety when a person is alcohol-free) (ICD-10—International Classification of Diseases 10th Revision; code F10.2—Mental and behavioral disorders due to use of alcohol), World Health Organization (WHO, 2010). As part of the treatment protocol, CATA provides outpatient and inpatient care. In inpatient care, the patients are voluntarily hospitalized for 20 days for alcoholic detoxification. All patients hospitalized during the period of this study were invited to participate on the first day of admission, regardless of the presence of gastrointestinal symptoms, and answered a questionnaire assessing socioeconomic aspects, some clinical conditions, and the pattern of alcohol intake. The questionnaire is provided as supplementary materials. The calculation of alcohol intake per day was performed according to Dufour, 1999 [16]. Patients who did not provide three fecal samples were excluded from this study.

The study was conducted in accordance with the Declaration of Helsinki, and the protocol was approved by the Research Ethics Committee of Nursery School, Federal University of Bahia, Brazil, under the registration number 367.464. All subjects gave their informed consent for inclusion before they participated in the study.

### 2.2. Parasitological Diagnosis 

Three fecal samples for each patient were evaluated by three different methods—spontaneous sedimentation [17], Baermann–Moraes [18], and agar plate culture (APC) [19]—at the Laboratory of Clinical and Toxicological Analysis of the Pharmacy College, Federal University of Bahia, Brazil. The parasite load was quantified through the number of larvae found in approximately 1 g of feces using the Baermann–Moraes method. The number of larvae was categorized as “non-quantified” when the parasite was not detected using the Baermann–Moraes method, 1–10, 11–50, 51–100, 101–500, and higher than 500 larvae/g of feces [20].

### 2.3. Statistical Analysis

Statistical analyses were performed using the Statistical Package for Social Science (SPSS), version 19.0 for Windows (SPSS Inc., Chicago, Illinois, USA) and Graph Pad Prism 5.0 (Graph Pad Software, Inc., San Diego, California, USA) software. To test the data normality assumption, the coefficients of skewness and kurtosis were calculated. A Gaussian distribution was considered when the coefficient of asymmetry presented values between –1 and +1. The quantitative variables were evaluated through descriptive measures (mean and standard deviation), while the semiquantitative variables were presented as frequency. Differences between means were evaluated using the unpaired *t*-test to compare two groups, while the differences in frequencies were measured using the chi square test. All tests were bicaudal, and the statistical significance was considered when p < 0.05. 

## 3. Results

This study included 1290 alcoholic patients, all male, with a mean age of 45.1 (standard deviation of 9.7) years. In the examination of the fecal samples, *S. stercoralis* was the most prevalent parasite, 14.5% (187/1290), followed by hookworm, 6.9% (87/1290), and *Schistosoma mansoni*, 3.5% (45/1290) (Table 1). 

Both infected and noninfected alcoholic patients did not present any statistically significant difference in relation to age, socioeconomic conditions, and sanitary aspects of residence. The subjects in both groups were from a low monthly-income bracket, with 70.7% (n = 814) receiving less than one minimum monthly Brazilian wage (approximately USD $200.00) and having a low educational level, with 77% (886/1151) studying until elementary school (Table 2). 

As with the socioeconomic characteristics, there was no difference in the drinking habits between infected and noninfected individuals, as a majority (87.1%; 1003/1151) drank alcoholic beverages every day. However, a difference was observed when comparing the percentage of individuals infected and noninfected with *S. stercoralis* who drank for up to 10 years, 22.9% (25/109) and 7.8% (55/710), respectively (p < 0.05) (Table 2). No differences were observed among individuals who drank for more than 21 years in both groups, 56.0 (61/109) and 53.4 (379/710), respectively (Table 2).

Alcohol intake was evaluated in 205 patients, with a mean of 428.7 ± 318.1 g/day, minimum and maximum of 24 and 1992 g/day, respectively. Alcoholic individuals infected with *Strongyloides stercoralis* (n = 39) had a higher daily consumption of alcohol than those who were not infected (n = 166), 528.6 and 403.0 g/day, respectively (p < 0.05). Individuals with a higher alcohol intake also presented a greater parasite load. Patients with ≤10 larvae/g of feces consumed a mean of 413.7 g/day, while individuals with >101 larvae/g of feces consumed 849.0 g/day (p < 0.05) (Table 3).

The mean parasite load of individuals infected with *Strongyloides stercoralis* was 69.9 ± 185.9 larvae/g of feces, ranging from non-quantified (when there were no larvae detected in the Baermann-Moraes method) to 1572 larvae/g of feces. Three patients presented more than 500 larvae/g of feces, which could indicate a hyperinfection due to the high parasite load. However, these patients only reported mild gastrointestinal symptoms. The parasitological method with the highest sensitivity was APC (97.9%; 183/187), when compared to the Baermann–Moraes (81.3%; 152/187) and spontaneous sedimentation (66.8%; 125/187) methods (p < 0.05). It was observed that the APC identified 33 positive samples that were negative in the Baermann–Moraes, with only one sample missing (Table 4). In samples with 1 to 10 larvae/g of feces, Baermann–Moraes and APC were the methods with the highest sensitivity, at 98.6% (69/70) and 95.7% (67/70), respectively. The spontaneous sedimentation method presented the lowest sensitivity, 62.9% (44/70). Both APC and Baermann–Moraes obtained 100% sensitivity when the parasite load was higher than 11 larvae/g of feces. Conversely, detection by spontaneous sedimentation only reached 100% sensitivity when there were more than 50 larvae/g of feces. Only one positive sample was diagnosed using the spontaneous sedimentation method alone (Table 4).

## 4. Discussion

*S. stercoralis* infection in immunocompromised patients can lead to a severe clinical condition, which has a high mortality rate. Our results confirm that, in endemic areas, *S. stercoralis* is the most common parasite found in alcoholic patients (14.5%; 187/1290). To our knowledge, this is the first work studying *S. stercoralis* infection in more than 1000 alcoholics. In Brazil, some authors have demonstrated that *S. stercoralis* infection prevalence in these individuals is approximately 20%, almost five times higher than in the general population [9,10,15]. The second most frequent parasite was hookworm, 6.9% (87/1290). The correlation between *S. stercoralis* and hookworms has been demonstrated by recent epidemiologic studies and highlights the common infectious route of these species, which is transmitted to the host, commonly, through larval penetration of intact skin [21,22]. Despite sharing the same routes of infection, it is worth mentioning that the frequency of hookworm is approximately half that of *S. stercoralis*.

Alcoholic individuals usually practice lower hygienic habits, and the consequence of this can be demonstrated by the elevated frequency of intestinal parasites [9,10,11]. In this study, most individuals lived in the state capital city, Salvador, and had access to a sewage system, paved streets, daily garbage collection, running water, and a bathroom at home. Nonetheless, most individuals had a low monthly income, 70.7% (814/1151), and presented a low educational level. In fact, most infected individuals had an elementary-school education, while individuals of the noninfected group had high school or college education. 

The number of years that each individual consumed alcohol seems to be associated with the frequency of parasite infection, considering the range of 0–10 years, which could be related to the changes in the immune system response in the early years of drinking. However, for both groups, most individuals were chronic alcoholics, for a period of more than 21 years, with a high amount of alcohol consumption. Oliveira et al., 2002, reported a higher prevalence of *Strongyloides* infection in alcoholic patients with liver cirrhosis than in alcoholic patients without this complication [23]. Although this observation has not been confirmed by other studies [9,24], it can be an indirect demonstration of a correlation between the severity of alcoholism and the prevalence of *Strongyloides* infection. In fact, alcoholic individuals infected with *S. stercoralis* presented a higher alcohol intake than non-infected alcoholic individuals. A similar result was found by Marques et al., 2010, where a positive correlation between the intensity of alcoholism and prevalence of the parasite in stools was demonstrated, regardless of the presence of liver cirrhosis [24]. However, Marques et al. did not evaluate the parasite load, only the frequency of *S. stercoralis* infection in alcoholic individuals, separated by alcohol intake. When alcohol consumption is categorized by the parasite load, it can be clearly observed that individuals with a higher larva excretion consume more alcohol. The increase in parasite load induced by large alcohol intake can be related to the high levels of cortisol, which can lead to an immune response impairment and/or elevation of the autoinfection cycle [24]. In either case, these individuals are more susceptible to severe strongyloidiasis. 

Because usually only larvae, but no eggs, are detected in stool samples, microscopic techniques that are often used in routine laboratories cannot diagnose *S. stercoralis* infection [25]. *S. stercoralis* diagnosis can be even more complicated in immunocompetent hosts, as the larval output is intermittent, and the parasite load is frequently low [8,20]. Therefore, a relatively high number of false-negative results can be found with conventional stool-based methods [26]. By contrast, immunocompromised individuals can present a distinctly higher parasite burden, which facilitates the diagnosis [20,27]. Geri et al., 2015 [7], reported a detection rate of 93% in both stool and sputum samples of 133 patients with hyperinfection. Additionally, in a previous study in our laboratory, it was demonstrated that for samples with more than 50 larvae/g of feces, all parasitological methods evaluated presented 100% sensitivity [20]. The same result was observed in this study with a larger number of samples.

Among parasitological techniques, the agar plate culture is the most sensitive method [20,28]. In this study, we were able to diagnose 33 samples that were undetected by the Baermann–Moraes method, which presented a high sensitivity even in individuals with a low parasite load. In comparison, the spontaneous sedimentation method only presented 100% sensitivity when the parasite load was higher than 50 larvae/g of feces. Hence, the accuracy of parasitological methods is directly associated to the number of larvae in the feces and even methods with low sensitivity, such as spontaneous sedimentation, can detect the presence of larvae in individuals with a high parasite load. This highlights the limitation in using the spontaneous sedimentation method in a laboratory routine, since the majority of *S. stercoralis* infected individuals have less than 10 larvae/g of feces [20]. While the APC method is more expensive, laborious, and time-consuming than conventional methods, it is still the best method available for parasitological diagnosis. 

The main limitation of this study was the inaccuracy in some patients’ responses related to the amount of alcohol consumption per day. During the application of questionnaires, many individuals had difficulties specifying the number of drinks and quantity-per-glass, or even day-to-day variations in drinking patterns. This inconsistency may be due to the application of questionnaires in the first day of hospitalization, when most of the patients presented withdrawal symptoms. Due to the doubtful responses from several patients about this topic, only conclusive answers were included in the study. We did not have access to information related to the presence of liver cirrhosis.

## 5. Conclusions

In our study, it was possible to observe that *S. stercoralis* is the most frequent parasite found in alcoholic individuals. The intensity of alcohol intake seems to be associated with *S. stercoralis* infection and a higher parasitic load, which could be potentially linked with severe strongyloidiasis. In addition, the APC should be used as the “gold standard” among the parasitological techniques, since it was the most sensitive method for *S. stercoralis* infection diagnosis, independently of the different ranges of parasite load. 

## Figures and Tables

**Table 1:** Frequency of enteroparasites in 1290 alcoholic patients attended at the Alcoholic Care and Treatment Center, Salvador, Bahia, Brazil.

Enteroparasites	Positivity % (n)
**Helminths**	
*Strongyloides stercoralis*	14.5 (187) *
Hookworm	6.9 (87)
*Schistosoma mansoni*	3.5 (45)
*Ascaris lumbricoides*	1.3 (17)
*Trichuris trichiura*	0.9 (11)
*Hymenolepis nana*	0.2 (2)
*Taenia* sp.	0.1 (1)
*Enterobius vermicularis*	0.1 (1)
**Protozoa**	
*Giardia duodenalis*	1.6 (20)
*Entamoeba histolytica/dispar/moshkovskii*	0.8 (10)
*Endolimax nana*	10.9 (141)
*Entamoeba coli*	2.8 (36)
*Iodamoeba butschilii*	0.6 (8)

* p < 0.05 compared to other pathogenic parasites identified.

**Table 2:** Demographic and socioeconomic characteristics, drinking patterns and signals and symptoms of alcoholic patients infected (n = 187) and noninfected (n = 964) with *S. stercoralis*.

Characteristics	*S. stercoralis* Infection		Total % (n)
	Positive % (n)	Negative% (n)	
**Age mean (years)**	44.8 ± 8.1	45.1 ± 9.9	45.1 ± 9.7
**Residence**			
Salvador	80.7 (151)	72.5 (699)	73.8 (850)
Other metropolitan cities	19.3 (36)	27.5 (265)	26.1 (301)
Tobacco smoker	70.0 (114)	60.8 (586)	60.8 (700)
**Education level**			
None	5.3 (10)	3.5 (34)	3.8 (44)
Elementary school	81.8 (153)	76.0 (733)	80.0 (886)
High school	12.3 (23)	18.7 (180)	17.6 (203)
College	0.5 (1)	1.7 (16)	1.5 (17)
Monthly income			
≤1 minimal wage ^†^	74.9 (140)	69.9 (674)	70.7 (814)
2 to 3 minimal wages	24.1 (45)	27.5 (265)	26.9 (310)
4 or more minimal wages ^‡^	0.1 (2)	2.6 (25)	2.3 (27)
**Sanitary conditions**			
None (homeless)	0.1 (2)	0.5 (5)	0.6 (7)
Sewage system	89.8 (168)	87.8 (846)	88.1(1014)
Paved streets	89.3 (167)	87.7 (845)	87.9 (1012)
Daily garbage collection	85.0 (159)	80.0 (770)	80.7 (929)
Potable water	96.8 (181)	94.5 (911)	94.8 (1092)
Bathroom at home	95.7 (179)	95.4 (920)	95.5 (1099)
**Drinking habits^●^**			
Everyday	87.7 (164)	87.0 (839)	87.1 (1003)
1 to 4 times/week	4.8 (9)	6.3 (61)	6.1 (70)
Over the weekend	6.9 (13)	5.8 (56)	6.0 (69)
1 to 3 times/month	0.5 (1)	0.7 (7)	0.7 (8)
**Alcohol intake (years) ^‡^ ▪**			
≤10	22.9 (25) *	7.8 (55)	9.7 (80)
11 to 20	21.1 (23)	38.9 (276)	36.5 (299)
>21	56.0 (61)	53.4 (379)	53.7 (440)
Type of drinks ^‡^			
Beer	15.6 (17)	13.9 (99)	14.2 (116)
Distilled	85.6 (90)	85.1 (604)	84.7 (694)
Other	1.8 (2)	0.7 (7)	1.1 (9)
**Signs and symptoms ^‡^**			
Diarrhea	9.2 (10)	9.2 (65)	9.2 (75)
Constipation	1.8 (2)	1.3 (9)	1.3 (11)
Abdominal pain	9.2 (10)	9.2 (65)	9.2 (75)
Nausea	0.9 (1)	1.7 (12)	1.6 (13)
Vomiting	2.8 (3)	4.8 (34)	4.5 (37)
Breathing Difficulties	1.8 (2)	0.3 (2)	0.5 (4)

^†^ Brazilian minimal wage is approximately USD $200.00. ^‡^ For these parameters, the number of patients analyzed was 109 for infected and 710 for non-infected alcoholic patients. * p < 0.05. ^●^ It refers to the drinking pattern of any alcohol beverage. ▪ This refers to how long the patient has been drinking alcohol, regardless of type and quantity.

**Table 3:** Alcohol intake in alcoholic patients infected (n = 39) and non-infected (n = 166) with *S. stercoralis* according to the parasite load.

Parasite Load (larvae/g of feces)	Number of Patients	Mean of Alcohol Intake (g/day)
Non-infected	166	403.0
<10	21	413.7
11–100	14	609.4
>100	4	849.0 *

* p < 0.05.

**Table 4:** Sensitivity of parasitological methods for *S. stercoralis* diagnosis according to the parasite load (n = 187).

Parasite Load (larva/gram of feces)	Sensitivity % (n)		
	Spontaneous Sedimentation	Baermann–Moraes	APC
Non-quantified ^†^	20.6 (7/34)	0.0 (0/34)	97.1 (33/34)
1–10	62.9 (44/70)	98.6 (69/70)	95.7 (67/70)
11–50	79.5 (35/44)	100.0 (44/44)	100.0 (44/44)
51–100	100.0 (17/17)	100.0 (17/17)	100.0(17/17)
101–500	100.0 (19/19)	100.0 (19/19)	100.0 (19/19)
>500	100.0 (3/3)	100.0 (3/3)	100.0 (3/3)
Total	66.8 (125/187)	81.3 (152/187)	97.9 (183/187) *

^†^ There were no larvae detected by the Baermann–Moraes method. * p < 0.05.

## Supplementary Materials

The following are available online at https://www.mdpi.com/2076-0817/9/6/422/s1, Questionnaire and Informed consent.
