# Supplementary material for: Strongyloides stercoralis in Alcoholic Patients: Implications of Alcohol Intake in the Frequency of Infection and Parasite Load
Source: Pathogens. 2020 May 28;9(6):422. doi: 10.3390/pathogens9060422 (PMC7350291; doi:10.3390/pathogens9060422)
Supplement: Supplementary file 1 [file pathogens-09-00422-s001.pdf]

UNIVERSIDADE FEDERAL DA BAHIA
FACULDADE DE FARMÁCIA
Departamento de Análises Clínicas e Toxicológicas
Laboratório de Pesquisa em Parasitologia
Tel.: (71) 3283-6950; 3283-6954

ID: ______________________
Infirmary / Bed:____________
OS: _____________________
Height: ____cm Weight: ____Kg

pName: ____ Phone:__________________

ID card______________________ Healthy card (SUS): ______________________ Date of birth: ____/___/____

Age: _______ Gender: ( ) M ( )F Marital status: ( )Single ( ) Married ( ) Civil union ( )Others____________

Mother's name: ____

Address: ____

- SOCIOECONOMIC AND SANITARY ASPECTS

1. What is your education level?

1. ( ) None
2. ( ) Incomplete elementary school
3. ( ) Complete elementary school
4. ( ) Incomplete high school
5. ( ) Complete high schoo
6. ( ) University education

2. What is the total family income (per month)?

1. ( ) <1 minimum wage
2. ( ) 2 minimum wages
3. ( ) 3 minimum wages
4. ( ) 4 minimum wages
5. ( ) > 4 minimum wages

3. Where does the water for drinking and cooking come from?

1. ( ) Tap water
2. ( ) Well
3. ( ) Tank
4. ( ) River
5. ( ) Dam
6. ( ) Others__________________

4. Boil or filter the water before drinking? ( ) Yes ( ) No ( ) Bottled water

5. Sanitary aspects:

Is there sewage in the place where you live? ( )Yes ( ) No
Do you have bathroom with toilet? ( )Yes ( ) No
Do you have a cesspool? ( )Yes ( ) No
Is there a hand sink in the bathroom? ( )Yes ( ) No
Are the streets paved? ( )Yes ( ) No

6. How is garbage collection done?

( ) All garbage is collected by the government
1. ( ) Daily
2. ( ) Weekly
3. ( ) None

( ) Other destinations
1. ( ) Trash is burnt
2. ( ) Garbage is accumulated in the streets
3. ( ) Garbage is accumulated in the yard

7. Do you walk barefoot? ( ) Yes ( ) No ( ) Sometimes

- SIGNS OR SYMPTOMS AT HOSPITAL ADMISSION:

1. Do you have any of the symptoms below?

1. ( ) Diarrhea
2. ( ) Intestinal constipation
3. ( ) Abdominal pain
4. ( ) Nausea
5. ( ) Vomiting
6. ( ) Breathing Problems
7. ( ) Others _________________

- SMOKE AND DRINK HABITS:

1. Do you currently smoke tobacco? ( ) Yes ( ) No ( ) Former smoker

- If you are a smoker or ex-smoker, how long do/did you smoke? ________________________

2. How long have you been drinking alcohol? ____

UNIVERSIDADE FEDERAL DA BAHIA
FACULDADE DE FARMÁCIA
Departamento de Análises Clínicas e Toxicológicas
Laboratório de Pesquisa em Parasitologia
Tel.: (71) 3283-6950; 3283-6954

3. How often do you usually drink alcohol?
(   ) Everyday (   ) 1-4 times/week (   ) Only on weekends
(   ) 1-3 times/month (   ) Less than once/month

4. What type of drink and how much do you usually consume per day?
(   ) Beer___________ (   ) Distilled___________ (   ) Others___________

*Please specify the type and number of drinks, type of glass used, and approximately the volume/quantity-per-glass, and if there is a day-to-day variation in drinking patterns.

____

____

DATE: _____/_____/_____ INTERVIEWER: ___________________ BLOOD COLLECTION TIME: _______
